# Snow patch refugia benefits for species of periglacial zones—Evidence from a high-elevation obligate

**DOI:** 10.1093/pnasnexus/pgad339

**Published:** 2023-11-07

**Authors:** Forest P Hayes, Joel Berger

**Affiliations:** Department of Fish, Wildlife, and Conservation Biology, Colorado State University, 951 Amy Van Dyken Way, Fort Collins, CO 80521, USA; Department of Fish, Wildlife, and Conservation Biology, Colorado State University, 951 Amy Van Dyken Way, Fort Collins, CO 80521, USA; Wildlife Conservation Society–Global Program, 2300 Southern Boulevard, Bronx, NY 10460, USA

**Keywords:** climate change, thermal stress, insect disturbance, snow, behavioral adaptation

## Abstract

Conserving Earth's most rapidly changing biomes necessitates understanding biological consequences of altered climes. Past species- and taxa-level responses to warming environs include numerous concentrated extirpations at the southern peripheries of distributions during the late Pleistocene. Less clear are localized capacities of cold-adapted species to mitigate thermal challenges against warming temperatures, especially through proximate behavioral and physiological adjustments. Whereas snow patches persist in periglacial zones and elsewhere, broad reductions in seasonal snow raise concerns about how and why species continue to use them. If snow patches play a functional role to combat increasing thermal demands, we predicted individuals would display an array of autonomic responses to increased temperatures modulated by wind, ambient temperature, and winter fur on and away from snow patches. We tested these predictions using a mammalian exemplar of high latitude and high elevation, mountain goats (*Oreamnos americanus*), using two sites in the northern Rocky Mountains, USA. Surprisingly, and contrary to expectations of reduced thermal stress, respiration rates were not decreased on snow patches but use of snow was strongly correlated with decreased metrics of insect harassment. As snow cover continues to decline in montane environs, the persistence of cold-adapted species depends on navigating concurrent changes in biotic communities and thermal environments and balancing competing pressures on behavioral and biological responses.

**Significance Statement**

Despite the strong associations between many taxa and cold environs, there remains great uncertainty about the biological benefits, if any, of using persistent snow during summer months. Contrary to the prevalent hypothesis that persistent snow provides thermal relief for cold-adapted species, we demonstrated that the use of snow patches facilitates insect avoidance and not thermoregulatory gains. While the duration and spatial extent of snow decline globally as the climate warms, its diminishing availability is likely to have substantive impacts on populations given a general pattern of associations between insects and temperatures at high elevations and latitudes.

## Introduction

**Fig. 1.**
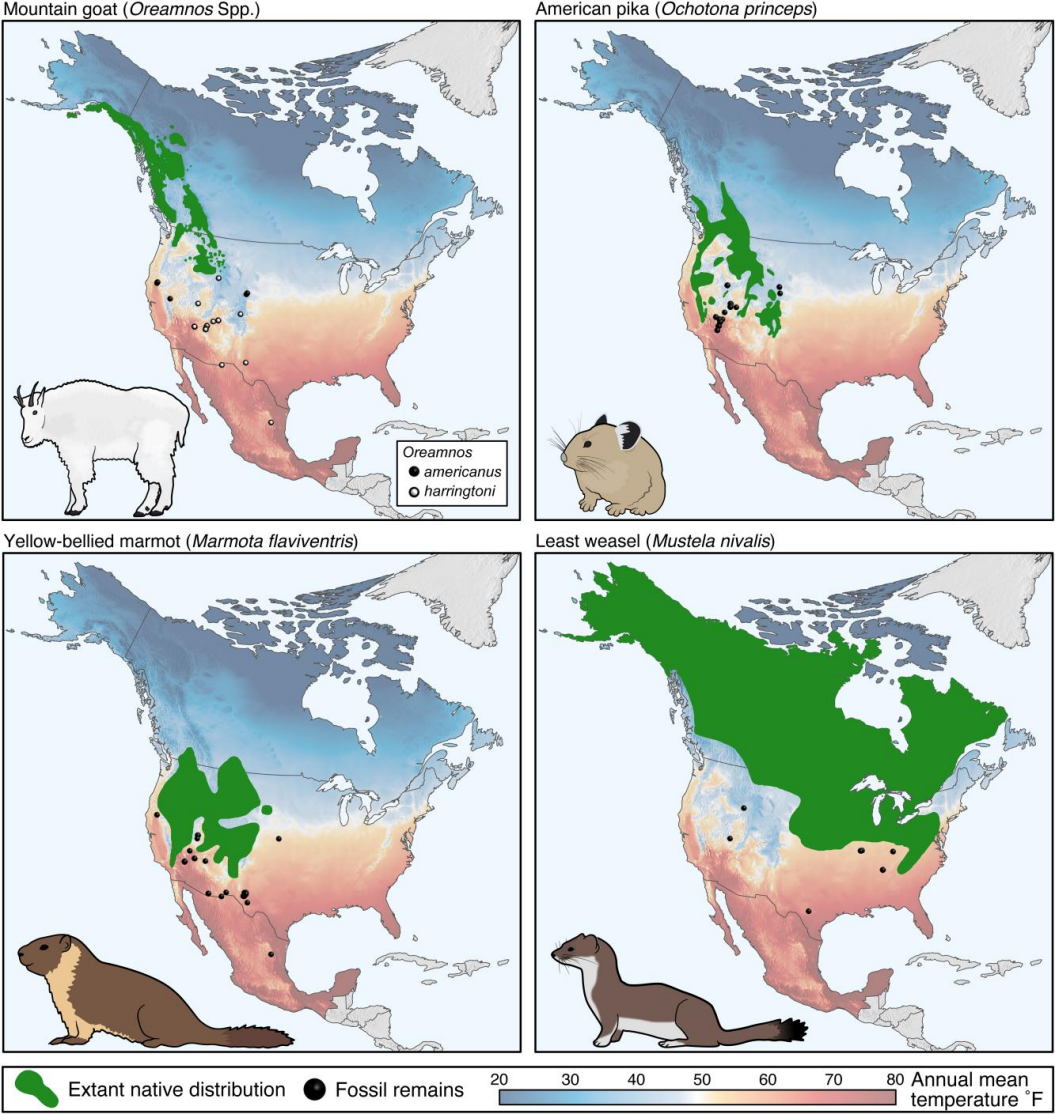
Extant native distribution of four exemplar cold-adapted species (mountain goat, American pika, yellow-bellied marmot, and least weasel) (6) and selected locales in which fossilized remains have been found (references provided in Table S1). Mean annual temperature (map shading) (7) illustrates that these species have been extirpated from warmer geographies but persist within cooler ecoregions.

High-elevation and high-latitude environments are warming at rates 2–5× faster than Earth's average (1), resulting in temperatures that are now reshaping ecological communities. Among many documented changes are upward shifts in vegetation communities and movements of invertebrates and vertebrates northward and to higher elevation (2–4). Historically, broad distributional shifts or site-specific extirpations in response to climatic change are not unusual and are reflected by Quaternary fossil deposits of cold-adapted mammals whose contemporary distributions are now situated at higher latitudes (Fig. 1) (5).

Climate-related warming has been a driving force in the truncation of many species along their lower latitude periphery, including those broadly associated with periglacial or cold climes such as mountain goats (*Oreamnos americanus*), pikas (*Ochotona* spp.), marmots (*Marmota* spp.), and lemmings (*Lemmus* spp.) (8). Others of Holarctic distribution, including moose (*Alces alces*) and wolverines (*Gulo gulo*), are less restricted yet still reliant on environments with snow and cold temperatures (9).

Despite the close association between species distributions and elements of climate, little is known about the near-term responses of chionophiles (i.e. organisms reliant on cold wintery conditions) to rapidly warming temperatures especially given the accentuated rates in mountainous and high-latitude environments (4). While direct threats from humans (e.g. habitat loss, overhunting, and nonnative species) are the principal risk for many species (10), warming temperatures are likely the paramount challenge for cold-adapted organisms (11). Individual animals feel the immediacy of these changes, wherein behavioral and physiological mechanisms may play fundamental roles in capacities to adjust (12).

Species of periglacial realms and of Earth's colder biomes are confronted by decreasing persistent snow (13). Yet, snow patches provide biological benefits to vertebrates (14), especially in summer (Table 1).

**Table 1.** The use of snow patches has been documented in numerous species, with varying perceived benefits and strengths of supporting evidence.

			Strength of evidence			
Benefit	Observed in		Mentioned	Empirical	Strong	Reference
Heat relief	Horse	*Equus caballus*	X			Keiper and Berger (15)
	Mountain goat	*Oreamnos americanus*		X		Sarmento et al. (16)
	Muskoxen	*Ovibos moschatus*	X			Hall (17)
	Caribou	*Rangifer tarandus*	X			Anderson and Nilssen (18)
	Grizzly bear	*Ursus arctos*	X			French et al. (19)
Insect relief	Horse	*Equus caballus*	X			Keiper and Berger (17)
	Mountain goat	*Oreamnos americanus*	X			Sarmento et al. (16)
	Caribou	*Rangifer tarandus*			X	Ion and Kershaw (20)
Water	Horse	*Equus caballus*	X			Keiper and Berger (15)
	Wild yak	*Bos grunniens mutus*		X		Berger et al. (21)
Play	Elk	*Cervus elaphus*	X			Berger (22)
	Horse	*Equus caballus*	X			Altmann (23)
	Bighorn sheep	*Ovis canadensis*	X			Berger (24)
	Wolverine	*Gulo gulo*	X			Polley (25)

Among these, grizzly bears (*Ursus arctos*) use snow-covered terrain for travel (19), and persistent ice or snow offers relief from heat across multiple mammalian orders (Table 1). Even during winter, remnant snow in arid regions of the Tibetan Plateau sustains lactating wild yaks (*Bos grunniens mutus*) (21).

**Fig. 2.**
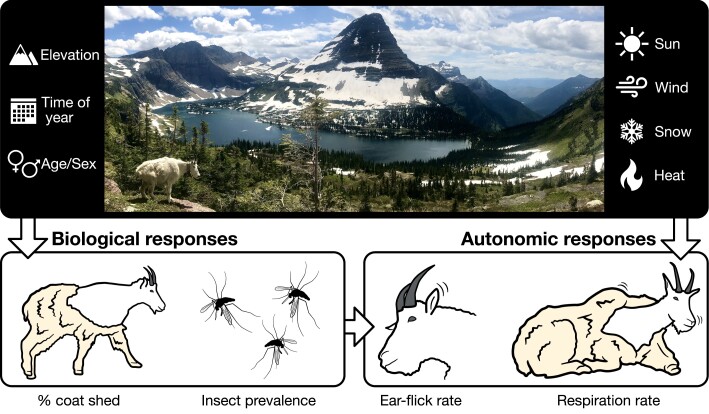
Overview of the primary study region (top photograph; Glacier National Park, 2020) and schematic of investigated variables we hypothesized to affect biological and autonomic responses. As depicted, biological responses are the amount of winter coat shed by a given individual and insect prevalence; autonomic responses are ear-flick and respiration rates. Time of year will, of course, be associated with snow and heat on a seasonal basis.

Given the twin challenges of increasing temperatures and broad declines in snow, a critical test for species in arctic, subarctic, and otherwise wintry temperate environs will be their ability to reduce thermal stress. On a coarse scale, options include geographic choices of locale and associated refuges (4). At finer scales are behavioral responses, including the use of persistent snow and ice (14), particularly because air over snow has lower ambient temperatures than above bare ground (20). Thus, we hypothesized that if snow patches provide thermal relief, individuals will exhibit a reduced respiration rate while on snow, and the use of snow patches during warm periods would be strongly incentivized (Fig. 2).

Alternatively, snow patches may be unimportant thermally but their use may benefit individuals by reducing disturbance from inimical insects. We rejected water as the primary importance of snow in our system as studies highlighting this are from arid environments (Table 1). Additionally, we did not evaluate play as a benefit of snow patches for, if this were the primary benefit, loss of snow patches would be unlikely to have demographic or distributional consequences. Herein, we evaluate two hypotheses of the benefit of snow patches: mitigation of thermal exposure and insect harassment.

To assess these hypotheses, we used in situ measurements of well-established proxies: respiration rate and ear-flick rate for thermal stress and ectoparasitic harassment, respectively. We focus on mountain goats, a cold-adapted, high-elevation mammal of western North America (26, 27). Our primary study area, Glacier National Park, nears the southern periphery of the species' extant native distribution (Fig. 1), where 85% of the glaciers have been lost since 1850 (28). Because of warming temperatures and the associated loss of glaciers, we believe our choice of study site and organism well serves as an exemplar to evaluate the potential benefits of snow patch use in other cold-reliant species (Fig. 2). To evaluate our hypotheses, we present data from both Glacier National Park and an introduced population at a higher elevation 1,000 km to the south in the Rocky Mountain cordillera (Mount Blue Sky, CO, USA).

Under continuing increases in global temperatures, species along the most vulnerable fronts are expected to experience the greatest stress (4). Nowhere might this be more notable in North America than the contemporary southern edges of the Rocky Mountains, where profound glacial loss and periglacial change are now occurring (1). With the local extinction of populations of species such as pika, yellow-bellied marmot, and least weasel from the southern edges of past distribution (Fig. 1), it appears that cold and snow are critical to the sustenance of chionophiles. While the consequences of global climate change will undoubtedly result in both net positive and net negative implications for different species, understanding individual components will improve our ability to predict outcomes for species reliant on snow. The results we report are an initial attempt to disentangle the role played by a single component of periglacial zones—snow patches—in the ecology of a cold-adapted obligate.

## Results

**Fig. 3.**
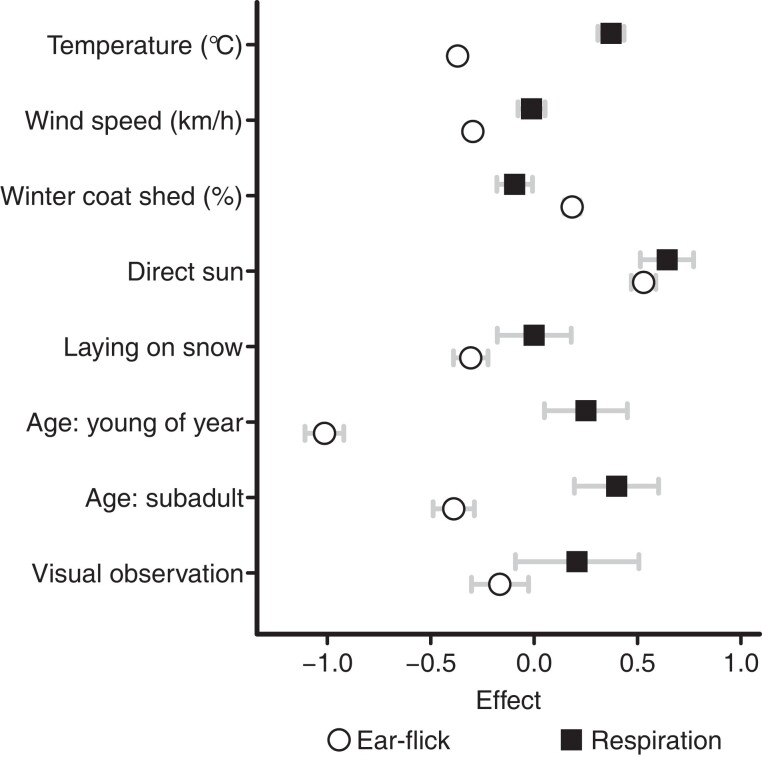
Effects of abiotic, weather, and biological variables (*y*-axis) on ear-flick and respiration rate of mountain goats from study sites in the Rocky Mountains, USA. Point estimates represent mean effect values, and whiskers show 95% credible intervals. The effects of continuous variables (winter coat shed and wind speed) are standardized to reflect a change of one standard deviation. Categorical variables (direct sun, lying on snow, age, visual observation) are additive effects with adult individuals and video observations treated as the intercept.

Our major hypothesis—that use of snow patches reduces thermal stress—was not supported based on measurements of respiration rate (Fig. 3 and Table S2).

**Fig. 4.**
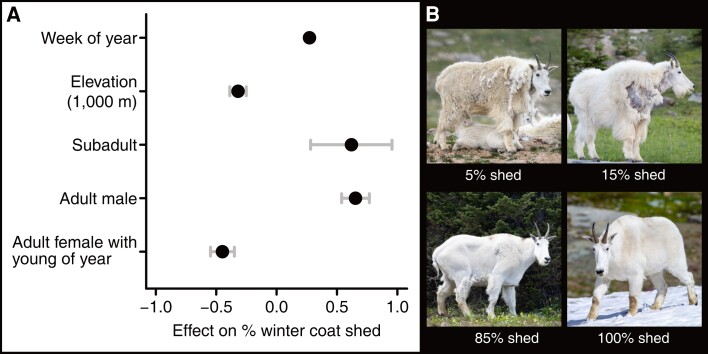
Spatiotemporal and biological relationships of shedding by mountain goats. A) Effects of week of year, elevation, and age class on percent of winter coat shed. Mean estimates represented by dots, and whiskers represent 95% credible intervals. The estimated effect for elevation represents an increase of 1,000 m. Categorical variables (age class and accompaniment by young of year) are additive effects with adult females (without young of year) treated as the intercept. B) Example of variation in mountain goat percent winter coat shed.

In contrast, the use of snow patches was strongly associated with reduced insect harassment. As a correlated variable, percent retention of the winter coat contributed to both respiration rate and autonomic behaviors associated with insect deterrence and notably mediated by wind. The positive association for direct sun and temperature with respiration rate (Fig. S1) provides key support for a foundational assumption of our study: that observed respiration rate is associated with increased thermal stress and that our field survey was able to capture this relationship. Percent of winter coat shed was negatively associated with elevation (Fig. 4 and Table S3) and negatively correlated with respiration rate (Fig. 3 and Table S2).

This suggests the timing of shedding may have a partial capacity to reduce thermal stress. The mean effect size was, however, small leading to a small biological effect (Fig. S1). Somewhat counterintuitively, lying on snow was not correlated with respiration rate, as reflected by the mean estimate of 0 and the credible interval equally split between positive and negative effects. Although thermoregulatory benefits of snow have previously been described for mountain goats (16), our greater sample size and improved methodology demonstrate a low probability of substantive thermal benefit. Perhaps the high albedo (i.e. reflectance) of snow increases animal exposure to solar radiation, offsetting and surpassing the cooling effects of snow (20).

With respect to hypothesized responses to ectoparasitic insects, percent winter coat shed and exposure to direct sun were positively associated with increased ear-flick rate (Fig. 3, Table S2, and Fig. S2). Both temperature and wind speed had very small credible intervals and were negatively correlated with ear-flick rate, with temperature having a greater effect. In strong contrast to respiration rate, recumbency on snow had a substantive negative effect on ear-flick rate. The wider credible interval for the effect of lying on snow (relative to other climatic variables) can be ascribed to the relatively small sample size. Younger age cohorts (young of year and subadult) were also negatively correlated with ear-flick rate, but with higher uncertainty in estimated effects due to a smaller sample size. Our results support the hypothesis that snow patch use is greatly incentivized for insect relief.

Percent of winter coat shed was strongly correlated with week of year, elevation, and age class (Fig. 4 and Table S3). Week of year was associated with decreased percent of coat shed with small variance in estimated effect and is well supported by prior literature (29, 30). An increase in elevation (of 1,000 m) was associated with a large negative effect on percent coat shed, suggesting winter coats are retained longer in cooler environs. Both subadult and adult male age classes were positively associated with shedding, while adult females accompanied by young of year retained their winter coats for longer. Prior research suggests the variability in estimated effects for age class is likely a result of individual condition (30).

Overall, our results contribute to a broader picture of a possible functional response to a presently declining resource—snow patches. While the hypothesis that snow patches provide thermal relief was not supported, insect harassment declined substantively when animals were recumbent on snow. Strong linkages between the impacts of ectoparasitic insects and demography (31) indicate that reduced capacity for insect avoidance has wide-ranging consequences for affected populations.

## Discussion

Changing climates have clear implications for species persistence as borne out by paleontological evidence. Where a general gradient of warming temperatures has progressed from lower latitude (warmest) to higher latitude (less so), species or population extirpations are prominent from their southern extents (Fig. 1). The ability of cold-adapted species to tolerate warm temperatures and associated biotic changes will be a key determinant of population trajectories and is a dominant concern for conservation planning (32). To date, many reported benefits of persistent snow are primarily observational or based on sparse empirical evidence (Table 1).

The results we offer represent a mixed picture for thermally sensitive species. On one hand, the use of snow patches does not appear to reduce thermal stress, at least through our metric of respiration rate, potentially limiting the opportunity for adaptative habitat use to mitigate increased heat. On the other hand, pika populations persist even in unexpected environments in part because they have adopted mechanisms to counter acute heat stress (33). Similar behavioral shifts to avoid increased temperature are well documented, with notable examples including moose and alpine ibex (34, 35), although physiological benefits are rarely quantified (Table 1). The trajectories of these species will be further influenced by climate-driven changes in resource availability and interspecific interactions which may have either positive or negative implications (36).

An important limitation of our study is that the use of opportunistic field observations precludes assessing the effect, if any, of the duration of time individuals spent on snow patches on our response variables. As an additional caveat, temperature and wind readings were collected at the observer's location. Due to variability at small spatial scales, perhaps these readings are only correlated to the conditions experienced by the observed individual. The imperfect correlation may have contributed to the seemingly counterintuitive decline in insect disturbance we noted at higher temperatures. As warming temperatures are creating more favorable conditions for insects, abundances are increasing even in areas where they were previously scarce and include a progressive expansion of activity in arctic and subarctic environs (2, 3). Accordingly, there are greater consequences of direct effects (e.g. blood loss) which can dramatically impact the body condition of mammals (31). The demographic consequences of this can be dramatic; for instance, increasing densities of winter ticks in some moose populations are responsible for >90% of calf mortality (37). Additionally, caribou are well known for their behavioral changes, including foregoing foraging and interrupted lactation, to avoid insects during summer months (31). Nevertheless, projected decreases in the availability of persistent snow and ice (38) will continue to challenge the extent to which species across geographically varied environments can use behavioral mechanisms and specific habitat components to evade insects.

Our result showing decreasing insect disturbance with increasing temperatures runs counter to patterns of insect activity (39). This underscores the complexity of faunal interactions. Further understanding of this relationship requires additional investigation to evaluate the possibility of differential thermal niches by insect species, improve the accuracy of weather measurements at the observed animal's location, and more thoroughly control for variability in conditions between the observer's and focal individual's location. While younger age classes were associated with decreased insect harassment and increased respiration rate relative to adults, this relationship is likely an underlying biological process associated with smaller body sizes and therefore unlikely to change as a direct function of climate.

Mountain goats, share much in common with other cold-adapted species such as wolverines, caribou, and pika. The degree of change being hoisted upon these species includes challenges of climate alteration, cryospheric loss, spread of disease, increasing ectoparasites, and changes in biotic communities (2, 8, 31). Changing climes are already exhibiting positive and negative effects on species in the arctic and subarctic environs, and the distributions of many species are expanding northward (2). At some point, mountain goats will colonize montane regions that become free of ice and snow, but in mid-Alaskan temperate environs, populations are projected to decline (27). Our data suggest declining availability of snow patches will most strongly impact mountain goats indirectly through reduced capacity to avoid inimical insects. This is but one component of the larger mosaic of climate challenges that must be understood to forecast population trajectories.

Our research on a species of the periglacial zones reemphasizes dynamic relationships between cold-adapted mammals and snow. Our findings indicate that the primary benefit of snow patch use (Table 1) for mountain goats is reduced insect disturbance and future declines in snow may expose them to greater risk. Additionally, concurrent changes in biotic communities and thermal environments may place competing pressures on behavioral and biological responses. The complexity of these interactions and the rapidity of current climate changes provide a clear mandate to understand impacts on today's southerly populations—lest our studies follow the recession of species to higher latitudes and altitudes.

## Methods

### Study site

Glacier National Park (48.8°N, −113.8°W) is a large (4,100 km^2^) federally managed and protected area characterized by high-elevation mountains (∼3,000 m) with alpine tundra and glacial lakes, and lower elevation (∼1,000 m) coniferous forests. Mountain goats are native to the region with an estimated population size of 2,000–3,000 in Montana (26). Predators included grizzly bears, black bears (*Ursus americanus*), mountain lions (*Puma concolor*), wolves, (*Canis lupus*), and coyotes (*Canis latrans*) (40).

Mount Blue Sky, Colorado (35.59°N, −105.64°W; 4,350 m) is located within a 300 km^2^ protected wilderness and characterized by alpine tundra, exposed rocky slopes, and coniferous forests at lower elevations. Mountain goats were introduced to Colorado by the Colorado Parks and Wildlife (formerly Game, Fish, and Parks Department) in 1948 and at Mount Blue Sky in 1961 where population estimates have since ranged from 60 to 200 individuals (41–43). Predators included black bears, mountain lions, and coyotes (44).

### Data collection

We used respiration rate, a physiological response to environmental heat stress (45, 46), to evaluate whether the use of snow patches had a cooling effect. Respiration rate is well suited to assessing changes in thermal environment as, under periods of heat stress, it is the first physiological mechanism used in many mammals to maintain body temperature (47). Dogs on warm days seek shade and breathe more rapidly. A study of domestic ruminants also reveals that respiration rates increase due to heightened temperatures (45). As such, measurements of respiration rate under variable conditions are one of the best methods for assessing the relative levels and immediacy of heat stress (46).

We used the ear-flick rate as a measure of insect disturbance (39). Prior research in which insect abundances were quantified indicates ear-flicks are positively correlated with measures of insect abundance (39) and is corroborated by decreased observations of ear-flicks in animals experimentally treated with insect repellents (48). We used relative levels of disturbance, as measured using ear-flick rates, to test the hypothesis that snow patch use provides refuge from insects. Avoidance of ectoparasitic insects is important as they have high direct costs in domestic and wild mammals, including disease transmission, blood loss, and redirection of feeding behavior (31, 39).

We recorded in situ observations of autonomic responses (respiration rate and ear-flick rate) of mountain goats and variation in local weather (Fig. 2) in Glacier National Park during May–September of 2020–2022 and at Mount Blue Sky during July of 2022. We recorded data using telephoto lenses and spotting scopes, typically at distances of 200–500 m, to minimize disturbance to animals. All observations were performed in accordance with protocols approved by the Institutional Animal Care and Use Committee of Colorado State University (IACUC #1067). We restricted observations to recumbent individuals to facilitate accurate measurement and to avoid confounding results because of additional energetic demands of standing or walking (49). Using the digitally recorded video observation, we counted the number of flank movements (45, 46) and the number of ear-flicks per ear during the survey period (39).

Under ideal conditions, visual observation of respiration rate and ear-flick rate may still be challenging and is prone to observation error and interobserver variation (46). To enhance repeatability and accuracy of measures, we captured video imagery of study animals with digital cameras. We used cameras with long telephoto lenses (Canon Inc, Tokyo, Japan, model: EOS 7D with EF 300 mm F/4L IS USM and Extender EF 1.4× II; EOS RP with RF 100–500 mm F4.5-7.1 L IS USM, and Extender RF 1.4×) to capture 15-s videos of bedded individuals. The 15-s measure of respiration is commonly used (46), as behavior (e.g. transition to standing) or local weather conditions (e.g. cloud cover) are unlikely to change during the survey period. We additionally collected a small number of visual observations (i.e. not digitally recorded) from experienced wildlife technicians following the same observation protocol and accounted for the possibility of increased observation error within the statistical model (described below).

For each observation, we classified each recumbent mountain goat by sex and age group (Fig. S3). We did not classify sex for juveniles as sexual dimorphism of mountain goats does not develop until the second summer (26). We also recorded the percent of winter coat shed for all individuals 1 year or older (Fig. S3), the latter variable under the assumption that percent of winter coat is associated with dissipation of body heat (29, 30). Further, we recorded two binary variables: exposure to direct sun and use of snow patches while recumbent. Last, we recorded the date, time, and geographic coordinates where the animal was observed to match observations with climatic and geographic variables. To reduce pseudoreplication, we collected repeat observations only after ≥15 min had elapsed.

We recorded temperature (°C) and wind speed (km/h) at 20-s intervals using a portable weather station (Nielsen-Kellerman Company, Boothwyn, PA, USA; model: Kestrel 5000 Environmental Meter) on a tripod at a height of 1 ft above the ground. The weather station was placed level and allowed to freely rotate, guided by a weathervane, to provide an accurate wind speed reading. These weather metrics were gathered at observer locations because it was either too disruptive to hike near goats at the time of data collection or not possible without extreme danger due to their occupancy of precipitous terrain. Our sample consisted of 928 in situ observations of mountain goats during 2020–2022 in Glacier National Park and at Mount Blue Sky (Table S4). Of these, digital video imagery accounted for >90% (*n=* 876).

### Analytical methods

#### Respiration and ear-flick rate

To link recorded temperature and wind metrics with observation data, we used the time of initiation for each observation (rounded down to minute) and calculated the average temperature and wind speed for that minute. We derived the number of ear-flicks/ear for the 15-s survey period by dividing the number of ear-flicks by the number of ears visible. We modeled respiration rate and ear-flick rates as independent response variables and centered and scaled continuous variables (e.g. percent shed, temperature, and wind speed) by subtracting their mean and dividing by their standard deviation to facilitate model fit and interpretation of results. We used a Bayesian framework for all models with 10,000 iterations and 5,000 iterations of burn in, and concluded models had converged when $\hat{R}$ < 1.01 (50). We analyzed both respiration and ear-flick rate as a function of local weather and biological condition using a generalized linear model:


$$\begin{array}{rl} Y_{\left(x1\ldots n\right)}∼ & \beta 0+\beta 1\times \mathrm{Coat}\;\mathrm{she}d_{\left(x1\ldots n\right)}\\ & +\beta 2\times \mathrm{Sun}\;\mathrm{exposur}e_{\left(x1\ldots n\right)}+\beta 3\times \mathrm{Temperatur}e_{\left(x1\ldots n\right)}\\ & +\beta 4\times \mathrm{Wind}\;\mathrm{spee}d_{\left(x1\ldots n\right)}+\beta 5\times \mathrm{Snow}\;\;\mathrm{us}e_{\left(x1\ldots n\right)}\\ & +\beta 6\times \mathrm{Age}\;\;\mathrm{clas}s_{\left(x1\ldots n\right)}+\beta 7\times \mathrm{Visual}\;\;\mathrm{observatio}n_{\left(x1\ldots n\right)} \end{array}$$

where *Y* represents the response variable (i.e. respiration rate or ear-flick rate), *x*1*…n* represents observation 1 through *n*, Coat shed was the percent of winter coat shed, Sun exposure was whether the majority of the goat was exposed to direct sunlight, Temperature was the ambient air temperature, Wind speed was the local wind speed (km/h), Snow use was a binary variable for indicating bedding on snow, Age was a categorical effect for young of year and subadult (i.e. 1–2 years old), and Visual observation was an additive effect for nondigital observations. Adult individuals (both male and female, age ≥3) are treated as the intercept. For ear-flick rate, we used a Poisson regression to account for overdispersion of count data (51). To incorporate information from observations without recorded temperature (*n=* 69) and wind data (*n=* 68), we interpolated unrecorded values by drawing samples from a distribution informed by observed data (51).

### Percent of winter coat shed

We derived the week of the year for each observation by calculating the number of 7-day periods elapsed between January 1 and the observation date. We used the recorded geographic coordinates of the observation to obtain elevation from the USGS National Elevation Dataset (USGS 2005). We then centered and scaled elevation to evaluate the effect a change of 1,000 m by subtracting the mean observation elevation and dividing by 1,000. To evaluate biological and geographic factors influencing percent of winter coat shed, we modeled shed as a linear model using a Bayesian framework:


$$\begin{array}{r} \mathrm{She}d_{\left(x1\ldots n\right)}∼\beta 0+\beta 1\times \mathrm{Elevatio}n_{\left(x1\ldots n\right)}+\beta 2\times \mathrm{Week}\;\mathrm{of}\;\mathrm{yea}r_{\left(x1\ldots n\right)}+\beta 3\\ \times \mathrm{Female}\;\mathrm{with}\;\mathrm{youn}g_{\left(x1\ldots n\right)}+\beta 4\times \mathrm{Age}/\mathrm{Se}x_{\left(x1\ldots n\right)} \end{array}$$

where Shed was the observed percent of winter coat shed, *x_1_…n* indicates each observation, Elevation was the elevation (m) of the observed individual, Week of year was a numeric count of elapsed 7-day periods since January 1, Female with young was an added effect for adult females accompanied by young of year, and Age/Sex was a categorical effect for young of year, juveniles (i.e. 1–2 years old), and adult males with adult females treated as the intercept.

## Supplementary Material

pgad339_Supplementary_Data

## Data Availability

All data and code supporting the findings of this manuscript are included as Supplementary material. Raw video files, from which observation data were derived, are not made available due to file sizes.
