# Supplementary material for: Snow patch refugia benefits for species of periglacial zones—Evidence from a high-elevation obligate
Source: PNAS Nexus. 2023 Nov 7;2(11):pgad339. doi: 10.1093/pnasnexus/pgad339 (PMC10635665; doi:10.1093/pnasnexus/pgad339)
Supplement: pgad339_Supplementary_Data [file pgad339_supplementary_data.zip › PNASNEXUS-PNASNEXUS-2023-00678R-s03.docx]

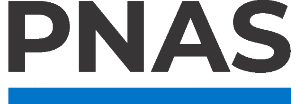


**Supporting Information for**

Snow patch refugia benefits for species of periglacial zones – evidence from a high elevation obligate

Forest P. Hayes and Joel Berger

Forest P. Hayes

Email: forest.hayes@colostate.edu

**This PDF file includes:**

Figures S1 to S3

Tables S1 to S4


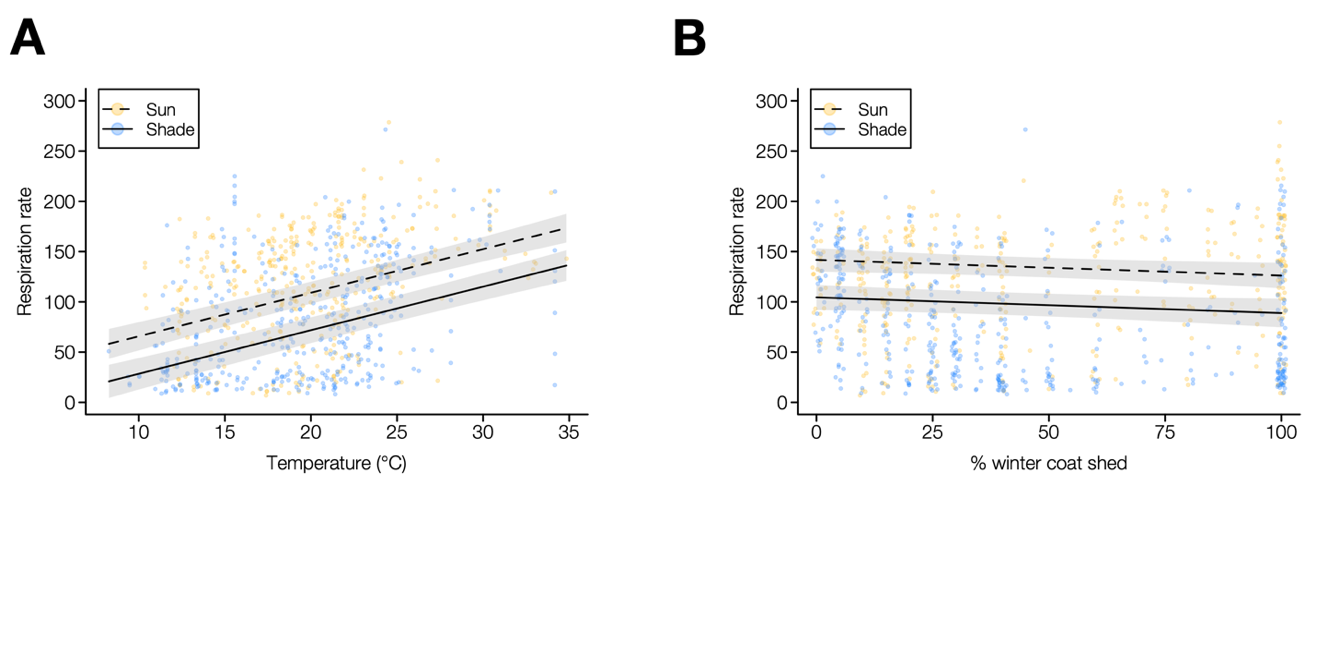
Fig. S1. Estimated biological effects of respiration rate in response to A) temperature and B) percent of winter coat shed. Points represent *in situ* measurements of respiration and explanatory variables. Lines represent modeled respiration response based on temperature and percent of winter coat in and out of direct sun, shaded regions represent 95% credible intervals.


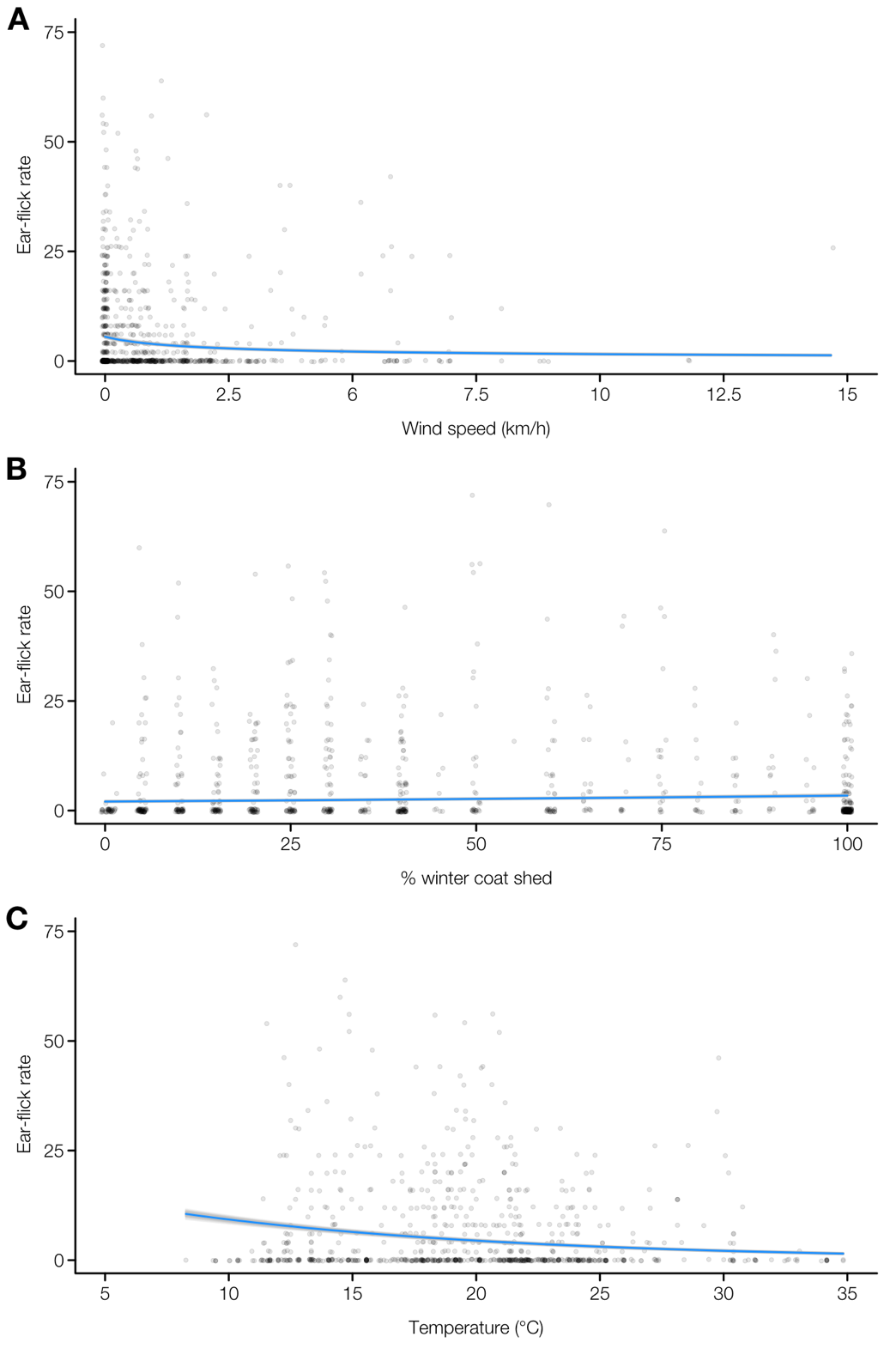


Fig. S2. Estimated biological effects of ear-flick rate (number of ear-flicks per ear per minute) in response to A) wind speed, B) percent of winter coat shed, and C) temperature. Points represent in situ measurements of ear-flick rate and explanatory variables. Lines represent modeled ear-flick response based on wind speed, % of winter coat shed, and temperature. Shaded regions represent 95% credible intervals.


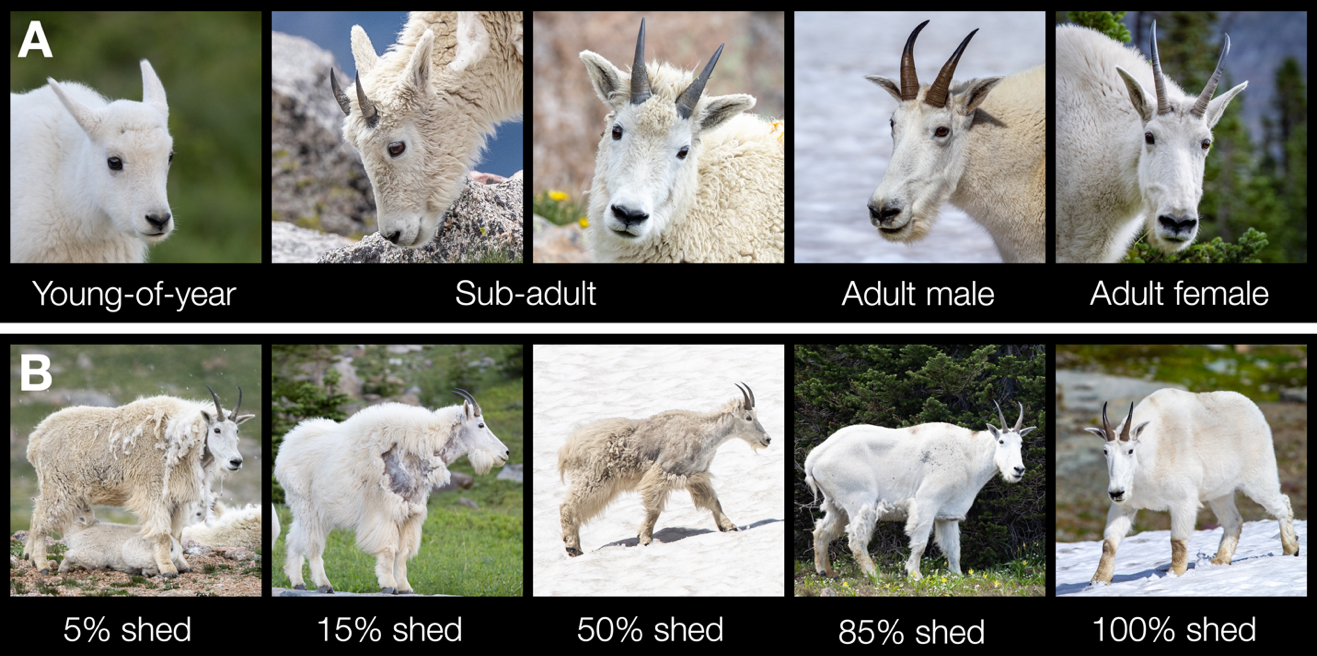


Fig. S3. Classification categories for age and sex of mountain goats. Individuals classified as either young-of-year (< 1 year old), sub-adult (1 or 2 years old), adult male (≥ 3 years old) or adult female (≥ 3 years old). B) Examples of estimated percent of winter coat shed.

Table S1. Information on fossil location for mountain goats (*Oreamnos americanus* and *Oreamnos harringtoni*), pika (*Ochotona princeps*), marmot (*Marmota flaviventris*) and weasel (*Mustela nivalis*). Site, geographic coordinates, age, and literature citations were obtained from the FAUNAP database (1).

| **Species** | **Site** | **Longitude** | **Latitude** | **Min age** | **Max age** | **Citation** |
| --- | --- | --- | --- | --- | --- | --- |
| *Oreamnos americanus* | Adrian Valley [LACM 6228] | -119.22194 | 39.15556 | 35000 | 400000 | Jefferson, G.T., H.G. McDonald, and S.D. Livingston. 2004. Catalogue of Late Quaternary and Holocene fossil vertebrates from Nevada. Occasional Papers 6. Nevada State Museum, Carson City, Nevada, USA. |
| *Oreamnos americanus* | Bell Cave [48AB304] | -105.36667 | 41.75 | 10000 | 13500 | Walker, D. N. 1987. Late Pleistocene/Holocene environmental changes in Wyoming: the mammalian record. Pages 334-393 in Late Quaternary mammalian biogeography and environments of the Great Plains and prairies (R. W. Graham, H. A. Semken, Jr., and M. A. Graham, editors), Illinois State Museum Scientific Papers 22. |
| *Oreamnos americanus* | Bell Cave [48AB304] | -105.36667 | 41.75 | 10000 | 13500 | Zeimans, G., and D. N. Walker. 1974. Bell Cave, Wyoming: preliminary archaeological and paleontological investigations. Wyoming Geological Survey, Report of Investigations 10:88-90. |
| *Oreamnos americanus* | Horned Owl Cave [48AB305] | -105.51667 | 41.56667 | 10000 | 35000 | Gebhard, D., D. A. Agogino, and V. Haynes. 1964. Horned Owl Cave, Wyoming. American Antiquity 29(3):360-368. |
| *Oreamnos americanus* | Horned Owl Cave [48AB305] | -105.51667 | 41.56667 | 10000 | 35000 | Guilday, J. E., H. W. Hamilton, and E. K. Adam. 1967. Animal remains from Horned Owl Cave, Albany County, Wyoming. Contributions to Geology, University of Wyoming 6(2):97-99. |
| *Oreamnos americanus* | Horned Owl Cave [48AB305] | -105.51667 | 41.56667 | 10000 | 35000 | Walker, D. N. 1987. Late Pleistocene/Holocene environmental changes in Wyoming: the mammalian record. Pages 334-393 in Late Quaternary mammalian biogeography and environments of the Great Plains and prairies (R. W. Graham, H. A. Semken, Jr., and M. A. Graham, editors), Illinois State Museum Scientific Papers 22. |
| *Oreamnos americanus* | Potter Creek Cave [5357,1055] | -122.28083 | 40.78389 | 11500 | 110000 | Kurtén, B.O.L, and E. Anderson. 1980. Pleistocene mammals of North America. Columbia University Press, New York, New York, USA. |
| *Oreamnos americanus* | Potter Creek Cave [5357,1055] | -122.28083 | 40.78389 | 11500 | 110000 | Lundelius, E. L., Jr., R. W. Graham, E. Anderson, J. Guilday, J. A. Holman, D. W. Steadman, and S. D. Webb. 1983. Terrestrial vertebrate faunas. Pages 311-353 in Late-Quaternary environments of the United States: volume 1, The late Pleistocene (S. C. Porter, editor), University of Minnesota Press, Minneapolis, Minnesota, USA. |
| *Oreamnos americanus* | Potter Creek Cave [5357,1055] | -122.28083 | 40.78389 | 11500 | 110000 | Stock, C. 1918. The Pleistocene fauna of Hawver Cave. University of California Publications, Bulletin of the Department of Geology 10(24):462-515. |
| *Oreamnos americanus* | Samwel Cave | -122.2318 | 40.9171 | 19063 | 25605 | Harris, A.H. 1985. Late Pleistocene Vertebrate Paleoecology of the West. University of Texas Press, Austin, Texas, USA. |
| *Oreamnos americanus* | Samwel Cave | -122.2318 | 40.9171 | 19063 | 25605 | Feranec, R.S., E.A. Hadley, J.L. Blois, A.D. Barnosky, and A. Paytan. 2007. Radiocarbon dates from the Pleistocene fossil deposits of Samwel Cave, Shasta County, California, USA. Radiocarbon 49(Nr 1):117-121. |
| *Oreamnos americanus* | Samwell Cave | -122.23787 | 40.91691 | 11500 | 110000 | Kurtén, B.O.L, and E. Anderson. 1980. Pleistocene mammals of North America. Columbia University Press, New York, New York, USA. |
| *Oreamnos americanus* | Samwell Cave | -122.23787 | 40.91691 | 11500 | 110000 | Furlong, E.L. 1906. The exploration of Samwel Cave. American Journal of Science 22:235-247. |
| *Oreamnos americanus* | Samwell Cave | -122.23787 | 40.91691 | 11500 | 110000 | Lundelius, E. L., Jr., R. W. Graham, E. Anderson, J. Guilday, J. A. Holman, D. W. Steadman, and S. D. Webb. 1983. Terrestrial vertebrate faunas. Pages 311-353 in Late-Quaternary environments of the United States: volume 1, The late Pleistocene (S. C. Porter, editor), University of Minnesota Press, Minneapolis, Minnesota, USA. |
| *Oreamnos americanus* | Samwell Cave | -122.23787 | 40.91691 | 11500 | 110000 | Payen, L. A., M. C. Hall, and M. D. Kelley. 1978. Radiocarbon and obsidian hydration studies of Samwell Cave. American Quaternary Association Abstracts, 5th Biennial Meeting, University Alberta, Edmonton. |
| *Oreamnos harringtoni* | Booth Canyon Local Fauna | -111.12889 | 43.29972 | 11800 | 29000 | Anderson, E. 1998. Quaternary wolverines (Gulo gulo) from Idaho. Pages 173-185 in W.A. Akerston, H.G. McDonald, D.J. Meldrum, and M.E.T. Flint, editors. And whereas ... Papers on the vertebrate paleontology of Idaho honoring John A. White, Volume 1. Occasional Paper 36. Idaho Museum of Natural History, Pocatello, Idaho, USA. |
| *Oreamnos harringtoni* | Booth Canyon Local Fauna | -111.12889 | 43.29972 | 11800 | 29000 | Mead, J.I., and M.C. Lawler. 1994. Skull, mandible, and metapodials of the extinct Harrington's mountain goat (Oreamnos harringtoni). Journal of Vertebrate Paleontology 14(4):562-576. |
| *Oreamnos harringtoni* | Booth Canyon Local Fauna | -111.12889 | 43.29972 | 11800 | 29000 | Akersten, W.A., S.J. Miller, and C.A. Repenning. 2002. The Booth Canyon Local Fauna, a depauperate mammalian assemblage from the Late Pleistocene of eastern Bonneville County, Idaho. Pages 101-130 in W.A. Akersten, M.E. Thompson, D.J. Meldrum, R.A. Rapp, and H.G. McDonald, editors. And Whereas‚Ä¶Papers on the Vertebrate Paleontology of Idaho Honoring John A. White, Volume 2. Occasional paper 37. Idaho Museum of Natural History, Pocatello, Idaho, USA. |
| *Oreamnos harringtoni* | Chuar Valley Midden 8b | -111.91667 | 36.18333 | 18000 | 19600 | Cole, K., and J.I. Mead. 1981. Late Quaternary animal remains from packrat middens in the eastern Grand Canyon, Arizona. Journal of the Arizona-Nevada Academy of Science 16:24-25. |
| *Oreamnos harringtoni* | Chuar Valley Midden 8b | -111.91667 | 36.18333 | 18000 | 19600 | Mead, J. I. 1981. The last 30,000 years of faunal history within the Grand Canyon, Arizona. Quaternary Research 15:311-326. |
| *Oreamnos harringtoni* | Hooper's Hollow [873] | -110.86667 | 37.25 | 10630 | 18840 | Mead, J. I., and L. D. Agenbroad. 1992. Isotope dating of Pleistocene dung deposits from the Colorado Plateau Arizona and Utah. Radiocarbon 34(1):1-19. |
| *Oreamnos harringtoni* | Muskox Cave | -104.5 | 32.11667 | 1 | 35000 | Harris, A. H. 1985. Late Pleistocene vertebrate paleoecology of the west. University of Texas Press, Austin. |
| *Oreamnos harringtoni* | Muskox Cave | -104.5 | 32.11667 | 1 | 35000 | Logan, L. E. 1981. The mammalian fossils of Muskox Cave, Eddy County, New Mexico. Proceedings Eighth International Congress of Speleology 1:159-160. |
| *Oreamnos harringtoni* | Muskox Cave | -104.5 | 32.11667 | 1 | 35000 | Jass, C. N., J. I. Mead, and L. E. Logan. 2000. Harrington's extinct mountain goat (Oreamnos harringtoni Stock 1936) from Muskox Cave, New Mexico. Texas Journal of Science 52(2):121-132. |
| *Oreamnos harringtoni* | Natural Bridges National Monument | -110 | 37.5 | 22000 | 25000 | Mead, J.I., L.D. Agenbroad, A.M. Phillips III, and L.T. Middleton. 1987. Extinct mountain goat (Oreamnos harringtoni) in southeastern Utah. Quaternary Research 27(3):323-331. [DOI: 10.1016/0033-5894(87)90087-1] |
| *Oreamnos harringtoni* | Rampart Cave | -113.93333 | 36.1 | 13000 | 11000 | Hansen, R. M. 1978. Shasta ground sloth food habits, Rampart Cave, Arizona. Paleobiology 4:302-319. |
| *Oreamnos harringtoni* | Rampart Cave | -113.93333 | 36.1 | 13000 | 11000 | Long, A., and P.S. Martin. 1974. Death of American ground sloths. Science 186:638-640.. |
| *Oreamnos harringtoni* | Rampart Cave | -113.93333 | 36.1 | 13000 | 11000 | Martin, P. S., B. E. Sabels, and D. Shutler, Jr. 1961. Rampart Cave coprolite and ecology of the Shasta ground sloth. American Journal of Science 259:102-127. |
| *Oreamnos harringtoni* | Rampart Cave | -113.93333 | 36.1 | 13000 | 11000 | Mead, J. I. 1981. The last 30,000 years of faunal history within the Grand Canyon, Arizona. Quaternary Research 15:311-326. |
| *Oreamnos harringtoni* | Rampart Cave | -113.93333 | 36.1 | 13000 | 11000 | Mead, J. I., L. D. Agenbroad, O. K. Davis, and P. S. Martin. 1986. Dung of Mammuthus in the arid southwest, North America. Quaternary Research 25:121-127. |
| *Oreamnos harringtoni* | Rampart Cave | -113.93333 | 36.1 | 13000 | 11000 | Van Devender, T. R., A. M. Phillips, III, and J. I. Mead. 1977. Late Pleistocene reptiles and mammals from the lower Grand Canyon of Arizona. Southwestern Naturalist 22:49-66. |
| *Oreamnos harringtoni* | Smith Creek Cave [26WP46] | -114.08333 | 39.33333 | 12600 | 35000 | Bryan, A. L. 1979. Smith Creek Cave. Pages 164-251 in The archaeology of Smith Creek Canyon, eastern Nevada (D. R. Tuohy and D. L. Rendall, editors), Nevada State Museum Anthropological Papers 17. |
| *Oreamnos harringtoni* | Smith Creek Cave [26WP46] | -114.08333 | 39.33333 | 12600 | 35000 | Mead, J.I., C.J. Bell, and L.K. Murray. 1992. Mictomys borealis (northern bog lemming) and the Wisconsin paleoecology of the east-central Great Basin. Quaternary Research 37(2):229-238. [DOI: 10.1016/0033-5894(92)90084-V] |
| *Oreamnos harringtoni* | Smith Creek Cave [26WP46] | -114.08333 | 39.33333 | 12600 | 35000 | Mead, J. I., R. S. Thompson, and T. R. Van Devender. 1982. Late Wisconsinan and Holocene fauna from Smith Creek Canyon, Snake River Range, Nevada. Transactions of the San Diego Society of Natural History 20(1):1-26. |
| *Oreamnos harringtoni* | Stanton's Cave [Ariz C:5:3] | -111.83333 | 36.5 | 1 | 17300 | Euler, R.C., editor. 1984. The archaeology, geology and paleobiology of Stanton's Cave. Grand Canyon Natural History Association Monograph 6. |
| *Oreamnos harringtoni* | Stanton's Cave [Ariz C:5:3] | -111.83333 | 36.5 | 1 | 17300 | Harington, C. R. 1984. Ungulate remains from Stanton's Cave and identification list. Pages 69-75 in The archaeology, geology, and paleobiology of Stanton's Cave (R. C. Euler, editor), Grand Canyon Natural History Association, Monograph No. 6. |
| *Oreamnos harringtoni* | Stanton's Cave [Ariz C:5:3] | -111.83333 | 36.5 | 1 | 17300 | Mead, J. I., L. D. Agenbroad, O. K. Davis, and P. S. Martin. 1986. Dung of Mammuthus in the arid southwest, North America. Quaternary Research 25:121-127. |
| *Oreamnos harringtoni* | Stanton's Cave [Ariz C:5:3] | -111.83333 | 36.5 | 1 | 17300 | Olsen, J. W., and S. J. Olsen. 1984. Zooarchaeological analysis of small vertebrates from Stanton's Cave, Arizona. Pages 49-57 in The archaeology, geology, and paleobiology of Stanton's Cave (R. C. Euler, editor), Grand Canyon Natural History Association, Monograph 6. |
| *Oreamnos harringtoni* | Stanton's Cave [Ariz C:5:3] | -111.83333 | 36.5 | 1 | 17300 | Robbins, E. I., P. S. Martin, and A. Long. 1984. Paleoecology of Stanton's Cave. Pages 115-130 in The archaeology, geology, and paleobiology of Stanton's Cave (R. C. Euler, editor), Grand Canyon Association Monograph 6. |
| *Oreamnos harringtoni* | Tse'an Bida Cave | -111.95 | 36 | 11000 | 13000 | Mead, J. I. 1981. The last 30,000 years of faunal history within the Grand Canyon, Arizona. Quaternary Research 15:311-326. |
| *Oreamnos harringtoni* | Tse'an Bida Cave | -111.95 | 36 | 11000 | 13000 | Mead, J. I., L. D. Agenbroad, O. K. Davis, and P. S. Martin. 1986. Dung of Mammuthus in the arid southwest, North America. Quaternary Research 25:121-127. |
| *Oreamnos harringtoni^*^* | San Josecito Cave | -99.91 | 23.95 | 11000 | 40000 | Jachowski, D., Kays, R., Butler, A., Hoylman, A. M., and Gompper, M. E. (2021). Tracking the decline of weasels in North America. PLoS ONE 16, e0254387. doi: 10.1371/journal.pone.0254387. |
| *Oreamnos harringtoni^*^* | U-Bar Cave | -108.4337 | 31.4745 | 25000 | 40000 | Harris, A. H. (1993). Quarternary vertebrates of New Mexico. *New Mexico Museum of Natural History and Science, Bulletin* 2, 179–198. |
| *Ochotona princeps* | Antelope Cave [SBC1.10.10] | -115.5 | 35.36667 | 10000 | 20000 | Jefferson, G.T. 1991. Rancholabrean age vertebrates from the Southeastern Mojave Desert, California. Pages 163-175 in R.E. Reynolds and J. Reynolds, editors. Crossing the borders: Quaternary studies in eastern California and Southwestern Nevada. Special Publication. San Bernardino County Museum Association, Redlands, California, USA. |
| *Ochotona princeps* | Antelope Cave [SBC1.10.10] | -115.5 | 35.36667 | 10000 | 20000 | Reynolds, R. E., R. L. Reynolds, C. J. Bell, and B. Pitzer. 1991. Vertebrate remains from Antelope Cave, Mescal Range, San Bernardino County, California. Pages 107-109 in Crossing the borders: Quaternary studies in eastern California and southwestern Nevada (R. E. Reynolds, editor), San Bernardino County Museum Association, Redlands. |
| *Ochotona princeps* | Arch Cave | -114.08333 | 39.11667 | 34040 | 34040 | Grayson, D.K. 1987. The biogeographic history of small mammals in the Great Basin: observations on the last 20,000 years. Journal of Mammalogy 68(2):359-375. |
| *Ochotona princeps* | Bell Cave [48AB304] | -105.36667 | 41.75 | 10000 | 13500 | Walker, D. N. 1987. Late Pleistocene/Holocene environmental changes in Wyoming: the mammalian record. Pages 334-393 in Late Quaternary mammalian biogeography and environments of the Great Plains and prairies (R. W. Graham, H. A. Semken, Jr., and M. A. Graham, editors), Illinois State Museum Scientific Papers 22. |
| *Ochotona princeps* | Bell Cave [48AB304] | -105.36667 | 41.75 | 10000 | 13500 | Zeimans, G., and D. N. Walker. 1974. Bell Cave, Wyoming: preliminary archaeological and paleontological investigations. Wyoming Geological Survey, Report of Investigations 10:88-90. |
| *Ochotona princeps* | Corn Creek Spring, Corn Creek PR 3, Las Vegas Valley | -115.35722 | 36.43917 | 13500 | 15100 | Jefferson, G.T., H.G. McDonald, and S.D. Livingston. 2004. Catalogue of Late Quaternary and Holocene fossil vertebrates from Nevada. Occasional Papers 6. Nevada State Museum, Carson City, Nevada, USA. |
| *Ochotona princeps* | Council Hall Cave [26WP229] | -114.16667 | 39.33333 | 4220 | 23900 | Bryan, A. L. 1979. Council Hall Cave. Pages 257-268 in The archaeology of Smith Creek Canyon, eastern Nevada (D. R. Tuohy and D. L. Rendall, editors), Nevada State Museum Anthropological Papers 17. |
| *Ochotona princeps* | Council Hall Cave [26WP229] | -114.16667 | 39.33333 | 4220 | 23900 | Mead, J. I., R. S. Thompson, and T. R. Van Devender. 1982. Late Wisconsinan and Holocene fauna from Smith Creek Canyon, Snake River Range, Nevada. Transactions of the San Diego Society of Natural History 20(1):1-26. |
| *Ochotona princeps* | Council Hall Cave [26WP229] | -114.16667 | 39.33333 | 4220 | 23900 | Miller, S. J. 1979. The archeological fauna of four sites in Smith Creek Canyon. Pages 273-329 in The archeology of Smith Creek Canyon, eastern Nevada (D. R. Tuohy and D. L. Randall, editors), Nevada State Museum Anthropological Papers 17. |
| *Ochotona princeps* | Council Hall Cave [26WP229] | -114.16667 | 39.33333 | 4220 | 23900 | Thompson, R. S., and J. I. Mead. 1982. Late Quaternary environments and biogeography in the Great Basin. Quaternary Research 17:39-55. |
| *Ochotona princeps* | Crystal Ball Cave | -113 | 39 | 10000 | 23000 | Heaton, T.H. 1985. Quaternary paleontology and paleoecology of Crystal Ball Cave, Millard County, Utah: with emphasis on the mammals and the description of a new species of fossil skunk. Great Basin Naturalist 45:337-390. |
| *Ochotona princeps* | Deer Creek Cave [EL-25] | -115.36667 | 41.75 | 10085 | 10085 | Heaton, T.H. 1985. Quaternary paleontology and paleoecology of Crystal Ball Cave, Millard County, Utah: with emphasis on the mammals and the description of a new species of fossil skunk. Great Basin Naturalist 45:337-390. |
| *Ochotona princeps* | Deer Creek Cave [EL-25] | -115.36667 | 41.75 | 10085 | 10085 | Ziegler, A. C. 1963. Unmodified mammal and bird remains from Deer Creek Cave, Elko County, Nevada. Pages 15-24 in Deer Creek Cave, Nevada (M. E. Shutler and R. Shutler, Jr., editors), Nevada State Museum Anthropological Papers 11. |
| *Ochotona princeps* | Eleana Range ER2-10 | -116.23417 | 37.12167 | 17100 | 17100 | Grayson, D.K. 1987. The biogeographic history of small mammals in the Great Basin: observations on the last 20,000 years. Journal of Mammalogy 68(2):359-375. |
| *Ochotona princeps* | Eleana Range ER2-11r | -116.23417 | 37.12167 | 10800 | 14310 | Grayson, D.K. 1987. The biogeographic history of small mammals in the Great Basin: observations on the last 20,000 years. Journal of Mammalogy 68(2):359-375. |
| *Ochotona princeps* | Eleana Range ER2-7 | -116.23417 | 37.12167 | 14760 | 14760 | Grayson, D.K. 1987. The biogeographic history of small mammals in the Great Basin: observations on the last 20,000 years. Journal of Mammalogy 68(2):359-375. |
| *Ochotona princeps* | Hendries Cave [26PLn5] | -114.87528 | 38.00028 | 1 | 71000 | Jefferson, G.T., H.G. McDonald, and S.D. Livingston. 2004. Catalogue of Late Quaternary and Holocene fossil vertebrates from Nevada. Occasional Papers 6. Nevada State Museum, Carson City, Nevada, USA. |
| *Ochotona princeps* | Kokoweef Cave [SBC1.11.13] | -115.5 | 35.41667 | 10000 | 20000 | Goodwin, H. T., and R. E. Reynolds. 1989. Late Quaternary Sciuridae from Kokoweef Cave, San Bernardino County, California. Bulletin of the Southern California Academy of Sciences 88(1):21-32. |
| *Ochotona princeps* | Kokoweef Cave [SBC1.11.13] | -115.5 | 35.41667 | 10000 | 20000 | Reynolds, R. E., R. L. Reynolds, C. J. Bell, N. J. Czaplewski, H. T. Goodwin, J. I. Mead, and B. Roth. 1991. The Kokoweef Cave faunal assemblage. Pages 97-103 in Crossing the borders: Quaternary studies in eastern California and southwestern Nevada (R. E. Reynolds, editor), San Bernardino County Museum Association, Redlands. |
| *Ochotona princeps* | Little Box Elder Cave [48CO287] | -105.61667 | 42.61667 | 9250 | 9250 | Anderson, E. 1968. Fauna of the Little Box Elder Cave, Converse County, Wyoming: the Carnivora. University of Colorado Studies Series in Earth Science 6. |
| *Ochotona princeps* | Little Box Elder Cave [48CO287] | -105.61667 | 42.61667 | 9250 | 9250 | Indeck, J. 1987. Sediment analysis and mammal faunal remains from Little Box Elder Cave, Wyoming. Ph.D. dissertation, University of Colorado, Boulder. |
| *Ochotona princeps* | Little Box Elder Cave [48CO287] | -105.61667 | 42.61667 | 9250 | 9250 | Walker, D. N. 1987. Late Pleistocene/Holocene environmental changes in Wyoming: the mammalian record. Pages 334-393 in Late Quaternary mammalian biogeography and environments of the Great Plains and prairies (R. W. Graham, H. A. Semken, Jr., and M. A. Graham, editors), Illinois State Museum Scientific Papers 22. |
| *Ochotona princeps* | Mescal Cave [SBC1.10.12] | -115.5 | 35.36667 | 10000 | 20000 | Brattstrom, B.H. 1958. New records of Cenozoic amphibians and reptiles from California. Bulletin of the Southern California Academy of Sciences 57:5-12. |
| *Ochotona princeps* | Mescal Cave [SBC1.10.12] | -115.5 | 35.36667 | 10000 | 20000 | Grayson, D.K. 1987. The biogeographic history of small mammals in the Great Basin: observations on the last 20,000 years. Journal of Mammalogy 68(2):359-375. |
| *Ochotona princeps* | Mescal Cave [SBC1.10.12] | -115.5 | 35.36667 | 10000 | 20000 | Jefferson, G.T. 1991. Rancholabrean age vertebrates from the Southeastern Mojave Desert, California. Pages 163-175 in R.E. Reynolds and J. Reynolds, editors. Crossing the borders: Quaternary studies in eastern California and Southwestern Nevada. Special Publication. San Bernardino County Museum Association, Redlands, California, USA. |
| *Ochotona princeps* | Mescal Cave [SBC1.10.12] | -115.5 | 35.36667 | 10000 | 20000 | Mehringer, P.J., Jr., and C.W. Ferguson. 1969. Pluvial occurrence of bristlecone pine (Pinus aristata) in a Mohave Desert mountain range. Journal of the Arizona Academy of Science 5(4):284-292. |
| *Ochotona princeps* | Mormon Mountain Cave [V6702A] | -115 | 37 | 8000 | 35000 | Jefferson, G. T. 1982. Late Pleistocene vertebrates from a Mormon Mountain cave in southern Nevada. Bulletin of the Southern California Academy of Sciences 81(3):121-127. |
| *Ochotona princeps* | Owl Cave 2 | -114.05 | 38.9 | 10000 | 35000 | Turnmire, K. L. 1987. An analysis of the mammalian fauna from Owl Cave One and Two, Snake Range, east central Nevada. Master's thesis, University of Maine, Orono. |
| *Ochotona princeps* | Pintwater Cave | -115.56139 | 36.94056 | 7285 | 32372 | Jefferson, G.T., H.G. McDonald, and S.D. Livingston. 2004. Catalogue of Late Quaternary and Holocene fossil vertebrates from Nevada. Occasional Papers 6. Nevada State Museum, Carson City, Nevada, USA. |
| *Ochotona princeps* | Pintwater Cave | -115.56139 | 36.94056 | 7285 | 32372 | Hockett, B.S. 2000. Paleobiogeographic changes at the Pleistocene-Holocene boundary near Pintwater Cave, southern Nevada. Quaternary Research 53:263-269. |
| *Ochotona princeps* | Potosi Mine Cave, Potosi Mountain 2A-2 Spring Range [1547] | -115.50028 | 35.96583 | 14050 | 15080 | Jefferson, G.T., H.G. McDonald, and S.D. Livingston. 2004. Catalogue of Late Quaternary and Holocene fossil vertebrates from Nevada. Occasional Papers 6. Nevada State Museum, Carson City, Nevada, USA. |
| *Ochotona princeps* | Potosi Mountain Midden 2 | -115.38333 | 36 | 12000 | 16500 | Mead, J. I., and L. K. Murray. 1991. Late Pleistocene vertebrates from the Potosi Mountain Packrat Midden, Spring Range, Nevada. Pages 124-126 in Crossing the borders: Quaternary studies in eastern California and southwestern Nevada (compiled by R. E. Reynolds), San Bernardino County Museum Association, Special Publication Redlands. |
| *Ochotona princeps* | Smith Creek Cave [26WP46] | -114.08333 | 39.33333 | 1 | 10000 | Bryan, A. L. 1979. Smith Creek Cave. Pages 164-251 in The archaeology of Smith Creek Canyon, eastern Nevada (D. R. Tuohy and D. L. Rendall, editors), Nevada State Museum Anthropological Papers 17. |
| *Ochotona princeps* | Smith Creek Cave [26WP46] | -114.08333 | 39.33333 | 1 | 10000 | Mead, J.I., C.J. Bell, and L.K. Murray. 1992. Mictomys borealis (northern bog lemming) and the Wisconsin paleoecology of the east-central Great Basin. Quaternary Research 37(2):229-238. [DOI: 10.1016/0033-5894(92)90084-V] |
| *Ochotona princeps* | Smith Creek Cave [26WP46] | -114.08333 | 39.33333 | 1 | 10000 | Mead, J. I., R. S. Thompson, and T. R. Van Devender. 1982. Late Wisconsinan and Holocene fauna from Smith Creek Canyon, Snake River Range, Nevada. Transactions of the San Diego Society of Natural History 20(1):1-26. |
| *Ochotona princeps* | Spires SP2 | -115.3 | 36.58333 | 18830 | 18830 | Grayson, D.K. 1987. The biogeographic history of small mammals in the Great Basin: observations on the last 20,000 years. Journal of Mammalogy 68(2):359-375. |
| *Marmota flaviventris* | Antelope Cave [SBC1.10.10] | -115.5 | 35.36667 | 10000 | 20000 | Jefferson, G.T. 1991. Rancholabrean age vertebrates from the Southeastern Mojave Desert, California. Pages 163-175 in R.E. Reynolds and J. Reynolds, editors. Crossing the borders: Quaternary studies in eastern California and Southwestern Nevada. Special Publication. San Bernardino County Museum Association, Redlands, California, USA. |
| *Marmota flaviventris* | Antelope Cave [SBC1.10.10] | -115.5 | 35.36667 | 10000 | 20000 | Reynolds, R. E., R. L. Reynolds, C. J. Bell, and B. Pitzer. 1991. Vertebrate remains from Antelope Cave, Mescal Range, San Bernardino County, California. Pages 107-109 in Crossing the borders: Quaternary studies in eastern California and southwestern Nevada (R. E. Reynolds, editor), San Bernardino County Museum Association, Redlands. |
| *Marmota flaviventris* | Baldy Peak Cave [MALB 29] | -107.75 | 32.11667 | 10000 | 35000 | Harris, A. H. 1985. Late Pleistocene vertebrate paleoecology of the west. University of Texas Press, Austin. |
| *Marmota flaviventris* | Burnet Cave | -104.75 | 32.36667 | 10900 | 11300 | Churcher, C. S. 1984. Sangamona: the furtive deer. Pages 316-331 in H. H. Genoways and M. R. Dawson, editors. Contributions in Quaternary vertebrate paleontology: a volume in memorial to John E. Guilday. Special Publication of Carnegie Museum of Natural History 8, Pittsburgh, Pennsylvania, USA. |
| *Marmota flaviventris* | Burnet Cave | -104.75 | 32.36667 | 10900 | 11300 | Harris, A. H. 1977. Wisconsin age environments in the northern Chihuahuan desert evidence from the higher vertebrates. Pages 23-52 in Transactions of the symposium on the biological resources of the Chihuahuan Desert Region, United States and Mexico (R. H. Wauer and D. H. Riskind, editors), National Park Service Transactions and Proceedings Series 3. |
| *Marmota flaviventris* | Burnet Cave | -104.75 | 32.36667 | 10900 | 11300 | Harris, A. H. 1985. Late Pleistocene vertebrate paleoecology of the west. University of Texas Press, Austin. |
| *Marmota flaviventris* | Burnet Cave | -104.75 | 32.36667 | 10900 | 11300 | Schultz, C. B., and E. B. Howard. 1935. The fauna of Burnet Cave, Guadalupe Mountains, New Mexico. Proceedings of the Academy of Natural Sciences of Philadelphia 87:273-298. |
| *Marmota flaviventris* | Council Hall Cave [26WP229] | -114.16667 | 39.33333 | 4220 | 23900 | Bryan, A. L. 1979. Council Hall Cave. Pages 257-268 in The archaeology of Smith Creek Canyon, eastern Nevada (D. R. Tuohy and D. L. Rendall, editors), Nevada State Museum Anthropological Papers 17. |
| *Marmota flaviventris* | Council Hall Cave [26WP229] | -114.16667 | 39.33333 | 4220 | 23900 | Mead, J. I., R. S. Thompson, and T. R. Van Devender. 1982. Late Wisconsinan and Holocene fauna from Smith Creek Canyon, Snake River Range, Nevada. Transactions of the San Diego Society of Natural History 20(1):1-26. |
| *Marmota flaviventris* | Council Hall Cave [26WP229] | -114.16667 | 39.33333 | 4220 | 23900 | Miller, S. J. 1979. The archeological fauna of four sites in Smith Creek Canyon. Pages 273-329 in The archeology of Smith Creek Canyon, eastern Nevada (D. R. Tuohy and D. L. Randall, editors), Nevada State Museum Anthropological Papers 17. |
| *Marmota flaviventris* | Council Hall Cave [26WP229] | -114.16667 | 39.33333 | 4220 | 23900 | Thompson, R. S., and J. I. Mead. 1982. Late Quaternary environments and biogeography in the Great Basin. Quaternary Research 17:39-55. |
| *Marmota flaviventris* | Dark Canyon Cave | -104.5 | 32.25 | 20120 | 25000 | Harris, A. H. 1977. Wisconsin age environments in the northern Chihuahuan desert evidence from the higher vertebrates. Pages 23-52 in Transactions of the symposium on the biological resources of the Chihuahuan Desert Region, United States and Mexico (R. H. Wauer and D. H. Riskind, editors), National Park Service Transactions and Proceedings Series 3. |
| *Marmota flaviventris* | Dark Canyon Cave | -104.5 | 32.25 | 20120 | 25000 | Harris, A. H. 1985. Late Pleistocene vertebrate paleoecology of the west. University of Texas Press, Austin. |
| *Marmota flaviventris* | Dry Cave | -104.48194 | 32.37361 | 13500 | 20000 | Harris, A. H. 1984. Two new species of late Pleistocene woodrats (Cricetidae: Neotoma) from New Mexico. Journal of Mammalogy 65:560-566. |
| *Marmota flaviventris* | Dry Cave | -104.48194 | 32.37361 | 13500 | 20000 | Harris, A. H. 1970. The Dry Cave mammalian fauna and late pluvial conditions in southeastern New Mexico. Texas Journal of Science 22(1):3-27. |
| *Marmota flaviventris* | Dry Cave | -104.48194 | 32.37361 | 13500 | 20000 | Harris, A. H. 1980. The paleontology of Dry Cave, New Mexico. National Geographic Society Research Report 12:331-338. |
| *Marmota flaviventris* | Dry Cave | -104.48194 | 32.37361 | 13500 | 20000 | Harris, A. H. 1985. Late Pleistocene vertebrate paleoecology of the west. University of Texas Press, Austin. |
| *Marmota flaviventris* | Dry Cave | -104.48194 | 32.37361 | 13500 | 20000 | Harris, A. H. 1987. Reconstruction of Mid-Wisconsin environments in southern New Mexico. National Geographic Research 3(2):142-151. |
| *Marmota flaviventris* | Dry Cave | -104.48194 | 32.37361 | 10730 | 14470 | Harris, A. H. 1984. Two new species of late Pleistocene woodrats (Cricetidae: Neotoma) from New Mexico. Journal of Mammalogy 65:560-566. |
| *Marmota flaviventris* | Dry Cave | -104.48194 | 32.37361 | 10730 | 14470 | Harris, A. H. 1970. The Dry Cave mammalian fauna and late pluvial conditions in southeastern New Mexico. Texas Journal of Science 22(1):3-27. |
| *Marmota flaviventris* | Dry Cave | -104.48194 | 32.37361 | 10730 | 14470 | Harris, A. H. 1980. The paleontology of Dry Cave, New Mexico. National Geographic Society Research Report 12:331-338. |
| *Marmota flaviventris* | Dry Cave | -104.48194 | 32.37361 | 10730 | 14470 | Harris, A. H. 1985. Late Pleistocene vertebrate paleoecology of the west. University of Texas Press, Austin. |
| *Marmota flaviventris* | Dry Cave | -104.48194 | 32.37361 | 10730 | 14470 | Harris, A. H. 1987. Reconstruction of Mid-Wisconsin environments in southern New Mexico. National Geographic Research 3(2):142-151. |
| *Marmota flaviventris* | Dust Cave [C-09] | -104.75 | 31.86667 | 13000 | 13000 | Harris, A. H. 1985. Late Pleistocene vertebrate paleoecology of the west. University of Texas Press, Austin. |
| *Marmota flaviventris* | Dust Cave [C-09] | -104.75 | 31.86667 | 13000 | 13000 | Van Devender, T.R., P.S. Martin, A.M. Phillips III, and W.G. Spaulding. 1975. Late Pleistocene biotic communities from the Guadalupe Mountains, Culberson County, Texas. Pages 107-113 in R.H. Wauer and D.H. Riskind, editors. Transactions of the Symposium on the Biological Resources of the Chihuahuan Desert Region United States and Mexico, Sul Ross State University, Alpine, Texas, 17-18 October 1974. United States Department of the Interior, National Park Service Transactions and Proceedings Series 3. |
| *Marmota flaviventris* | Fowlkes Cave | -104.11667 | 31 | 10000 | 35000 | Dalquest, W. W., and F. B. Stangl, Jr. 1984. Late Pleistocene and early Recent mammals from Fowlkes Cave, southern Culberson County, Texas. Pages 432-455 in Contributions in Quaternary vertebrate paleontology: a volume in memorial to John E. Guilday (H. H. Genoways and M. R. Dawson, editors), Carnegie Museum of Natural History Special Publications 8. |
| *Marmota flaviventris* | Fowlkes Cave | -104.11667 | 31 | 10000 | 35000 | Dalquest, W. W., and F. B. Stangl, Jr. 1986. Post-Pleistocene mammals of the Apache Mountains, Culberson County, Texas, with comments on zoogeography of the Trans-Pecos Front Range. Occasional Papers of the Museum of Texas Tech University 104:2-35. |
| *Marmota flaviventris* | Kokoweef Cave [SBC1.11.13] | -115.5 | 35.41667 | 10000 | 20000 | Goodwin, H. T., and R. E. Reynolds. 1989. Late Quaternary Sciuridae from Kokoweef Cave, San Bernardino County, California. Bulletin of the Southern California Academy of Sciences 88(1):21-32. |
| *Marmota flaviventris* | Kokoweef Cave [SBC1.11.13] | -115.5 | 35.41667 | 10000 | 20000 | Reynolds, R. E., R. L. Reynolds, C. J. Bell, N. J. Czaplewski, H. T. Goodwin, J. I. Mead, and B. Roth. 1991. The Kokoweef Cave faunal assemblage. Pages 97-103 in Crossing the borders: Quaternary studies in eastern California and southwestern Nevada (R. E. Reynolds, editor), San Bernardino County Museum Association, Redlands. |
| *Marmota flaviventris* | Lower Sloth Cave | -104.86667 | 31.86667 | 1 | 11590 | Harris, A. H. 1985. Late Pleistocene vertebrate paleoecology of the west. University of Texas Press, Austin. |
| *Marmota flaviventris* | Lower Sloth Cave | -104.86667 | 31.86667 | 1 | 11590 | Logan, L. E. 1983. Paleoecological implications of the mammalian fauna of Lower Sloth Cave Guadalupe Mountains, Texas. National Speleological Society Bulletin 45:3-11. |
| *Marmota flaviventris* | Lower Sloth Cave | -104.86667 | 31.86667 | 1 | 11590 | Van Devender, T.R., P.S. Martin, A.M. Phillips III, and W.G. Spaulding. 1975. Late Pleistocene biotic communities from the Guadalupe Mountains, Culberson County, Texas. Pages 107-113 in R.H. Wauer and D.H. Riskind, editors. Transactions of the Symposium on the Biological Resources of the Chihuahuan Desert Region United States and Mexico, Sul Ross State University, Alpine, Texas, 17-18 October 1974. United States Department of the Interior, National Park Service Transactions and Proceedings Series 3. |
| *Marmota flaviventris* | Mormon Mountain Cave [V6702A] | -115 | 37 | 8000 | 35000 | Jefferson, G. T. 1982. Late Pleistocene vertebrates from a Mormon Mountain cave in southern Nevada. Bulletin of the Southern California Academy of Sciences 81(3):121-127. |
| *Marmota flaviventris* | Muskox Cave | -104.5 | 32.11667 | 1 | 35000 | Harris, A. H. 1985. Late Pleistocene vertebrate paleoecology of the west. University of Texas Press, Austin. |
| *Marmota flaviventris* | Muskox Cave | -104.5 | 32.11667 | 1 | 35000 | Logan, L. E. 1981. The mammalian fossils of Muskox Cave, Eddy County, New Mexico. Proceedings Eighth International Congress of Speleology 1:159-160. |
| *Marmota flaviventris* | Muskox Cave | -104.5 | 32.11667 | 1 | 35000 | Jass, C. N., J. I. Mead, and L. E. Logan. 2000. Harrington's extinct mountain goat (Oreamnos harringtoni Stock 1936) from Muskox Cave, New Mexico. Texas Journal of Science 52(2):121-132. |
| *Marmota flaviventris* | North Cove [25HN164] | -99.36667 | 40.11667 | 12965 | 14700 | Stewart, J. D. 1987. Prehistoric and historic cultural resources of selected sites at Harlan County Lake, Harlan County, Nebraska. U.S. Army Corps of Engineers, Final Report, Kansas City. |
| *Marmota flaviventris* | Papago Springs Cave | -110.61667 | 31.61667 | 1 | 1 | Czaplewski, N. J., J. I. Mead, T. L. Ku, and L. D. Agenbroad. 1989. Radiometric age assignment for Papago Springs Cave deposits, southeastern Arizona. Southwestern Naturalist 34(2):278-281. |
| *Marmota flaviventris* | Papago Springs Cave | -110.61667 | 31.61667 | 1 | 1 | Harris, A. H. 1985. Late Pleistocene vertebrate paleoecology of the west. University of Texas Press, Austin. |
| *Marmota flaviventris* | Papago Springs Cave | -110.61667 | 31.61667 | 1 | 1 | Lindsay, E.H. 1978. Late Cenozoic vertebrate faunas, southeastern Arizona. Pages 269-275 in J.F. Callender, J.C. Wilt, R.E. Clemons, and H.L. James, editors. Land of Cochise (southeastern Arizona). New Mexico Geological Society\r\r\nGuidebook. New Mexico Geological Society, Socorro, New Mexico, USA. |
| *Marmota flaviventris* | Papago Springs Cave | -110.61667 | 31.61667 | 1 | 1 | Skinner, M.F. 1942. The fauna of Papago Springs Cave, Arizona, and a study of Stockoceros; with three new antilocaprines from Nebraska and Arizona. Bulletion of the American Museum of Natural History 80(6):143-220. |
| *Marmota flaviventris* | Papago Springs Cave | -110.61667 | 31.61667 | 1 | 1 | Czaplewski, N. J., W. D. Peachey, J. I. Mead, T.-L. Ku, and C. J. Bell. 1999. Papago Springs Cave revisited part I; geologic setting, cave deposits, and radiometric dates. Occasional Papers of the Oklahoma Museum of Natural History 3:1-25. |
| *Marmota flaviventris* | Papago Springs Cave | -110.61667 | 31.61667 | 1 | 1 | Czaplewski, N.J., J.I. Mead, C.J. Bell, W.D. Peachey, and T.-L. Ku. 1999. Papago Springs Cave revisited, part II: vertebrate paleofauna. Occasional Papers of the Oklahoma Museum of Natural History 5:1-41. |
| *Marmota flaviventris* | Papago Springs Cave | -110.61667 | 31.61667 | 31000 | 110000 | Czaplewski, N. J., J. I. Mead, T. L. Ku, and L. D. Agenbroad. 1989. Radiometric age assignment for Papago Springs Cave deposits, southeastern Arizona. Southwestern Naturalist 34(2):278-281. |
| *Marmota flaviventris* | Papago Springs Cave | -110.61667 | 31.61667 | 31000 | 110000 | Harris, A. H. 1985. Late Pleistocene vertebrate paleoecology of the west. University of Texas Press, Austin. |
| *Marmota flaviventris* | Papago Springs Cave | -110.61667 | 31.61667 | 31000 | 110000 | Lindsay, E.H. 1978. Late Cenozoic vertebrate faunas, southeastern Arizona. Pages 269-275 in J.F. Callender, J.C. Wilt, R.E. Clemons, and H.L. James, editors. Land of Cochise (southeastern Arizona). New Mexico Geological Society\r\r\nGuidebook. New Mexico Geological Society, Socorro, New Mexico, USA. |
| *Marmota flaviventris* | Papago Springs Cave | -110.61667 | 31.61667 | 31000 | 110000 | Skinner, M.F. 1942. The fauna of Papago Springs Cave, Arizona, and a study of Stockoceros; with three new antilocaprines from Nebraska and Arizona. Bulletion of the American Museum of Natural History 80(6):143-220. |
| *Marmota flaviventris* | Papago Springs Cave | -110.61667 | 31.61667 | 31000 | 110000 | Czaplewski, N. J., W. D. Peachey, J. I. Mead, T.-L. Ku, and C. J. Bell. 1999. Papago Springs Cave revisited part I; geologic setting, cave deposits, and radiometric dates. Occasional Papers of the Oklahoma Museum of Natural History 3:1-25. |
| *Marmota flaviventris* | Papago Springs Cave | -110.61667 | 31.61667 | 31000 | 110000 | Czaplewski, N.J., J.I. Mead, C.J. Bell, W.D. Peachey, and T.-L. Ku. 1999. Papago Springs Cave revisited, part II: vertebrate paleofauna. Occasional Papers of the Oklahoma Museum of Natural History 5:1-41. |
| *Marmota flaviventris* | Potter Creek Cave [5357,1055] | -122.28083 | 40.78389 | 11500 | 110000 | Kurtén, B.O.L, and E. Anderson. 1980. Pleistocene mammals of North America. Columbia University Press, New York, New York, USA. |
| *Marmota flaviventris* | Potter Creek Cave [5357,1055] | -122.28083 | 40.78389 | 11500 | 110000 | Lundelius, E. L., Jr., R. W. Graham, E. Anderson, J. Guilday, J. A. Holman, D. W. Steadman, and S. D. Webb. 1983. Terrestrial vertebrate faunas. Pages 311-353 in Late-Quaternary environments of the United States: volume 1, The late Pleistocene (S. C. Porter, editor), University of Minnesota Press, Minneapolis, Minnesota, USA. |
| *Marmota flaviventris* | Potter Creek Cave [5357,1055] | -122.28083 | 40.78389 | 11500 | 110000 | Stock, C. 1918. The Pleistocene fauna of Hawver Cave. University of California Publications, Bulletin of the Department of Geology 10(24):462-515. |
| *Marmota flaviventris* | Pratt Cave [TMM-41172] | -104.75 | 31.86667 | 1420 | 2820 | Lundelius, E. L., Jr. 1979. Post-Pleistocene mammals from Pratt Cave and their environmental significance. Pages 239-257 in Biological investigations in the Guadalupe Mountains National Park, Texas, (H. H. Genoways and R. J. Baker, editors), National Park Service, Proceedings and Transactions Series 4. |
| *Marmota flaviventris* | Rampart Cave | -113.93333 | 36.1 | 10000 | 13000 | Hansen, R. M. 1978. Shasta ground sloth food habits, Rampart Cave, Arizona. Paleobiology 4:302-319. |
| *Marmota flaviventris* | Rampart Cave | -113.93333 | 36.1 | 10000 | 13000 | Long, A., and P.S. Martin. 1974. Death of American ground sloths. Science 186:638-640.. |
| *Marmota flaviventris* | Rampart Cave | -113.93333 | 36.1 | 10000 | 13000 | Martin, P. S., B. E. Sabels, and D. Shutler, Jr. 1961. Rampart Cave coprolite and ecology of the Shasta ground sloth. American Journal of Science 259:102-127. |
| *Marmota flaviventris* | Rampart Cave | -113.93333 | 36.1 | 10000 | 13000 | Mead, J. I. 1981. The last 30,000 years of faunal history within the Grand Canyon, Arizona. Quaternary Research 15:311-326. |
| *Marmota flaviventris* | Rampart Cave | -113.93333 | 36.1 | 10000 | 13000 | Mead, J. I., L. D. Agenbroad, O. K. Davis, and P. S. Martin. 1986. Dung of Mammuthus in the arid southwest, North America. Quaternary Research 25:121-127. |
| *Marmota flaviventris* | Rampart Cave | -113.93333 | 36.1 | 10000 | 13000 | Van Devender, T. R., A. M. Phillips, III, and J. I. Mead. 1977. Late Pleistocene reptiles and mammals from the lower Grand Canyon of Arizona. Southwestern Naturalist 22:49-66. |
| *Marmota flaviventris* | Smith Creek Cave [26WP46] | -114.08333 | 39.33333 | 9280 | 12150 | Bryan, A. L. 1979. Smith Creek Cave. Pages 164-251 in The archaeology of Smith Creek Canyon, eastern Nevada (D. R. Tuohy and D. L. Rendall, editors), Nevada State Museum Anthropological Papers 17. |
| *Marmota flaviventris* | Smith Creek Cave [26WP46] | -114.08333 | 39.33333 | 9280 | 12150 | Mead, J.I., C.J. Bell, and L.K. Murray. 1992. Mictomys borealis (northern bog lemming) and the Wisconsin paleoecology of the east-central Great Basin. Quaternary Research 37(2):229-238. [DOI: 10.1016/0033-5894(92)90084-V] |
| *Marmota flaviventris* | Smith Creek Cave [26WP46] | -114.08333 | 39.33333 | 9280 | 12150 | Mead, J. I., R. S. Thompson, and T. R. Van Devender. 1982. Late Wisconsinan and Holocene fauna from Smith Creek Canyon, Snake River Range, Nevada. Transactions of the San Diego Society of Natural History 20(1):1-26. |
| *Marmota flaviventris* | Snake Creek Burial Cave | -114.11667 | 38.86667 | 10000 | 35000 | Heaton, T. H. 1987. Initial investigation of vertebrate remains from Snake Creek Burial Cave, White Pine County, Nevada. Current Research in the Pleistocene 4:107-109. |
| *Marmota flaviventris* | Snake Creek Burial Cave | -114.11667 | 38.86667 | 10000 | 35000 | Mead, E. M., and J. I. Mead. 1989. Snake Creek Burial Cave and a review of the Quaternary mustelids of the Great Basin. Great Basin Naturalist 49(2):143-154. |
| *Marmota flaviventris* | Tse'An Kaetan Cave-Grand Canyon | -112 | 36 | 14220 | 30600 | Agenbroad, L. D., and J. I. Mead. 1987. Late Pleistocene alluvium and megafauna dung deposits of the central Colorado Plateau. Pages 68-84 in G. H. Davis and E. M. VandenDolder, editors. Geologic diversity of Arizona and its margins: excursions to choice areas Arizona Bureau of Geology and Mineral Technology Special Paper 5. |
| *Marmota flaviventris* | Tse'An Kaetan Cave-Grand Canyon | -112 | 36 | 14220 | 30600 | Emslie, S. 1987. Age and diet of fossil California condors in Grand Canyon, Arizona. Science 237:768-770. |
| *Marmota flaviventris* | Tse'An Kaetan Cave-Grand Canyon | -112 | 36 | 14220 | 30600 | Mead, J. I., and L. D. Agenbroad. 1992. Isotope dating of Pleistocene dung deposits from the Colorado Plateau Arizona and Utah. Radiocarbon 34(1):1-19. |
| *Marmota flaviventris* | U-Bar Cave [LA5689] | -108.4337 | 31.4745 | 25000 | 40000 | Bense, J. A. 1972. Cascade phase: a study in the effect of the altithermal on a cultural system. Ph.D. dissertation, Washington State University, Pullman. |
| *Marmota flaviventris* | U-Bar Cave [LA5689] | -108.4337 | 31.4745 | 25000 | 40000 | Harris, A. H. 1985. Late Pleistocene vertebrate paleoecology of the west. University of Texas Press, Austin. |
| *Marmota flaviventris* | U-Bar Cave [LA5689] | -108.4337 | 31.4745 | 25000 | 40000 | Harris, A. H. 1987. Reconstruction of Mid-Wisconsin environments in southern New Mexico. National Geographic Research 3(2):142-151. |
| *Marmota flaviventris* | Upper Sloth Cave [TTu-Tex-2] | -104.75 | 31.86667 | 11000 | 11760 | Harris, A. H. 1985. Late Pleistocene vertebrate paleoecology of the west. University of Texas Press, Austin. |
| *Marmota flaviventris* | Upper Sloth Cave [TTu-Tex-2] | -104.75 | 31.86667 | 11000 | 11760 | Logan, L. E., and C. C. Black. 1979. The Quaternary vertebrate fauna of Upper Sloth Cave, Guadalupe Mountains National Park, Texas. Pages 141-158 in Biological investigations in the Guadalupe Mountains National Park, Texas (H. H. Genoways and R. J. Baker, editors), National Park Service, Proceedings and Transactions Series 4. |
| *Marmota flaviventris* | Upper Sloth Cave [TTu-Tex-2] | -104.75 | 31.86667 | 11000 | 11760 | Van Devender, T.R., P.S. Martin, A.M. Phillips III, and W.G. Spaulding. 1975. Late Pleistocene biotic communities from the Guadalupe Mountains, Culberson County, Texas. Pages 107-113 in R.H. Wauer and D.H. Riskind, editors. Transactions of the Symposium on the Biological Resources of the Chihuahuan Desert Region United States and Mexico, Sul Ross State University, Alpine, Texas, 17-18 October 1974. United States Department of the Interior, National Park Service Transactions and Proceedings Series 3. |
| *Marmota flaviventris* | Vulture Cave | -113.93333 | 36.1 | 1 | 13500 | Mead, J.I., and A.M. Phillips III. 1981. The late Pleistocene and Holocene fauna and flora of Vulture Cave, Grand Canyon, Arizona. Southwestern Naturalist 26:257-288. |
| *Marmota flaviventris** | San Josecito Cave | -99.91 | 23.95 | 11000 | 40000 | Jachowski, D., Kays, R., Butler, A., Hoylman, A. M., and Gompper, M. E. (2021). Tracking the decline of weasels in North America. PLoS ONE 16, e0254387. doi: 10.1371/journal.pone.0254387. |
| *Mustela nivalis* | Cheek Bend Cave [40MU261] | -86.86667 | 35.5 | 12500 | 16500 | Klippel, W.E., and P.W. Parmalee. 1982. The paleontology of Cheek Bend Cave, Maury County, Tennessee: phase II report. Report to the Tennessee Valley Authority. |
| *Mustela nivalis* | Crankshaft Cave | -90.5 | 38.36667 | 11500 | 110000 | Parmalee, P.W., R.D. Oesch, and J.E. Guilday. 1969. Pleistocene and recent vertebrate faunas from Crankshaft Cave, Missouri. Report of Investigations 14. Illinois State Museum, Springfield, Illinois. |
| *Mustela nivalis* | Meyer Cave | -90.25389 | 38.39667 | 1 | 11450 | Parmalee, P.W. 1967. A recent cave bone deposit in southwestern Illinois. National Speleological Society Bulletin 29(4):119-147. |
| *Mustela nivalis* | Moonshiner | -112.61667 | 43.36667 | 8000 | 10000 | Kurtén, B.O.L, and E. Anderson. 1980. Pleistocene mammals of North America. Columbia University Press, New York, New York, USA. |
| *Mustela nivalis* | Moonshiner | -112.61667 | 43.36667 | 8000 | 10000 | Mullican, T. R., and L. N. Carraway. 1990. Shrew remains from Moonshiner and Middle Butte caves, Idaho. Journal of Mammalogy 71(3):351-356. |
| *Mustela nivalis* | Moonshiner | -112.61667 | 43.36667 | 8000 | 10000 | White, J. A., H. G. McDonald, E. Anderson, and J. M. Soiset. 1984. Lava blisters as carnivore traps. Pages 241-256 in Contributions in Quaternary vertebrate paleontology: a volume in memorial to John E. Guilday (H. H. Genoways and M. R. Dawson, editors), Carnegie Museum of Natural History Special Publications 8. |
| *Mustela nivalis* | Snake Creek Burial Cave | -114.11667 | 38.86667 | 10000 | 35000 | Heaton, T. H. 1987. Initial investigation of vertebrate remains from Snake Creek Burial Cave, White Pine County, Nevada. Current Research in the Pleistocene 4:107-109. |
| *Mustela nivalis* | Snake Creek Burial Cave | -114.11667 | 38.86667 | 10000 | 35000 | Mead, E. M., and J. I. Mead. 1989. Snake Creek Burial Cave and a review of the Quaternary mustelids of the Great Basin. Great Basin Naturalist 49(2):143-154. |
| *Mustela nivalis* | Welsh Cave [15WD13] | -84.74722 | 37.87361 | 12950 | 12950 | Guilday, J. E., H. W. Hamilton, and A. D. McCrady. 1971. The Welsh Cave peccaries (Platygonus) and associated fauna, Kentucky Pleistocene. Annals of the Carnegie Museum 43:249-320. |
| *Mustela nivalis** | Hall's Cave | -99.42 | 30.05 | 14700 | 20000 | Seersholm, F. V., Werndly, D. J., Grealy, A., Johnson, T., Keenan Early, E. M., Lundelius, E. L., et al. (2020). Rapid range shifts and megafaunal extinctions associated with late Pleistocene climate change. Nat Commun 11, 2770. doi: 10.1038/s41467-020-16502-3. |

*These data were separately acquired and not included in the FAUNMAP database.

Table S2. Numeric summary of the effects of biological condition and local weather on mountain goat respiration rate (breaths/minute) and ear-flick rate (flicks/ear/minute) of mountain goats in Glacier National Park, USA, during 2020–2022 and at Mount Blue Sky, CO, USA.

|  | **Effect on respiration rate** | | | |  | **Effect on ear-flick rate** | | | |
| --- | --- | --- | --- | --- | --- | --- | --- | --- | --- |
|  | **Mean** | **SD** | **2.5%** | **97.5%** |  | **Mean** | **SD** | **2.5%** | **97.5%** |
| Temperature (°C) | 0.37 | 0.03 | 0.31 | 0.43 |  | –0.37 | 0.01 | –0.40 | –0.34 |
| Wind speed (km/h) | ­–0.01 | 0.03 | –0.08 | 0.05 |  | –0.30 | 0.01 | –0.32 | –0.27 |
| Winter coat shed (%) | –0.09 | 0.04 | –0.18 | -0.01 |  | 0.18 | 0.02 | 0.15 | 0.22 |
| Direct sun | 0.64 | 0.07 | 0.51 | 0.77 |  | 0.53 | 0.03 | 0.47 | 0.59 |
| Laying on snow | 0.00 | 0.09 | –0.18 | 0.18 |  | –0.31 | 0.04 | –0.39 | –0.22 |
| Age: young-of-year | 0.25 | 0.10 | 0.05 | 0.45 |  | –1.01 | 0.05 | –1.11 | –0.92 |
| Age: sub-adult | 0.40 | 0.10 | 0.19 | 0.60 |  | –0.39 | 0.05 | –0.49 | –0.29 |
| Visual observation | 0.21 | 0.15 | –0.09 | 0.51 |  | –0.17 | 0.07 | –0.30 | –0.03 |

Table S3. Numeric summary of the effects of biological condition and local weather on percent of winter coat shed by mountain goats in Glacier National Park, USA, during 2020–2022 and at Mount Blue Sky, CO, USA.

|  | **Mean** | **SD** | **2.5%** | **97.5%** |
| --- | --- | --- | --- | --- |
| Week of year | 0.27 | 0.01 | 0.26 | 0.29 |
| Elevation (1,000 m) | –0.32 | 0.04 | –0.39 | –0.25 |
| Sub-adult | 0.62 | 0.18 | 0.28 | 0.96 |
| Adult male | 0.65 | 0.06 | 0.54 | 0.77 |
| Adult female with young-of-year | –0.45 | 0.05 | –0.55 | –0.35 |

Table S4. Numeric summary of mountain goat observations by year on and off snow in Glacier National Park, USA, during 2020–2022 and at Mount Blue Sky, CO, USA during July of 2022.

|  | **Glacier National Park** | | |  | **Mount Blue Sky** | |
| --- | --- | --- | --- | --- | --- | --- |
|  | **2020** | **2021** | **2022** |  | **2022** | **Total** |
| On snow | 19 | 128 | 5 |  | 0 | 152 |
| Not on snow | 329 | 268 | 92 |  | 87 | 776 |
| Total | 348 | 396 | 97 |  | 87 | 928 |

**SI References**

1. R. W. Graham, E. L. Lundelius, *Faunmap: A Database Documenting Late Quaternary Distributions of Mammal Species in the United States* (Illinois State Museum, 1994).
